# Mixed-Mode Dynamic Stress Intensity Factors and Fracture Analysis Using Ordinary State-Based Peridynamics

**DOI:** 10.3390/ma19122560

**Published:** 2026-06-12

**Authors:** Yanyun Ru, Fei Li, Xingyu Li, Caidan Wang, Qianlong Yang, Shuqin Zheng, Lei Zhou, Xu Wang

**Affiliations:** 1 School of Civil Engineering, Chongqing Jiaotong University, Chongqing 400074, China; ruyy@cqjtu.edu.cn (Y.R.); 622240035002@mails.cqjtu.edu.cn (F.L.); 622250030015@mails.cqjtu.edu.cn (X.L.); 622250030010@mails.cqjtu.edu.cn (C.W.); 17318401832@163.com (Q.Y.); xuwang@cqjtu.edu.cn (X.W.); 2 Key Laboratory of Intelligent Lifeline Protection and Emergency Technology for Resident ATY, Wenzhou University of Technology, Wenzhou 325035, China

**Keywords:** mixed-mode fracture, peridynamics, dynamic stress intensity factors, circular hole, crack propagation, brittle solids

## Abstract

An ordinary state-based peridynamic (OSPD) approach combined with an interaction integral method is proposed to calculate dynamic stress intensity factors (DSIFs) and simulate crack propagation in two-dimensional cracked brittle solids. Numerical investigations are carried out for mode I and mixed-mode cracked plates under static, quasi-static, and dynamic loading conditions. A local damping scheme is incorporated into the peridynamic equations of motion to achieve convergence in static and quasi-static analyses. The influence of circular holes on DSIFs and crack propagation paths is systematically examined. Quantitative analyses of elastic deformation and quasi-static fracture behavior for mode I and mixed-mode cracks are verified through the uniaxial tension of a slab. The peak values of DSIFs exceed their static counterparts under dynamic loading. Complex dynamic fracture phenomena, including crack branching in both straight and inclined edge cracks, are successfully captured. The results obtained by the OSPD approach are validated through comparisons with theoretical benchmarks and finite element results, demonstrating high accuracy and effectiveness in calculating elastic deformation and stress intensity factors (SIFs), as well as accurately predicting crack propagation paths in quasi-static and dynamic fracture problems in brittle solids. Beyond the benchmark problems, the proposed OSPD approach is particularly well-suited for investigating more complex fracture systems.

## 1. Introduction

Brittle fractures represent a major failure mode in engineering materials and structures, threatening structural integrity due to their sudden and catastrophic nature. Therefore, understanding crack propagation in brittle solids is essential across various engineering fields. However, accurate prediction of these processes remains challenging in solid mechanics, particularly when complex cracks are present, such as branching and multiple crack interactions.

In classical continuum mechanics, materials are treated as continuums during deformation, and spatial partial derivatives are used to solve boundary value problems. In contrast, damage and failure analysis often involves discontinuities such as cracks and interfaces. Across these discontinuous surfaces, the derivatives of the displacement field become undefined or ill-suited, rendering the derivation of spatial differential equations difficult within the conventional framework. Various extended and enriched numerical methods have been proposed to overcome these limitations, such as the Extended Finite Element Method (XFEM) [1,2], the Cohesive Zone Method (CZM) [3,4], the Boundary Element Method (BEM) [5,6], the Meshfree Method (MFM) [7,8], and the Phase-Field Method (PFM) [9,10]. Although these special techniques offer several advantages for solving fracture problems, some challenges, such as dynamic fracture, multiple cracks, and fragmentation, remain in fracture modeling.

Peridynamics (PD), introduced by Silling [11,12], is a nonlocal continuum theory that avoids classical continuum mechanics’ difficulties with discontinuities like cracks. In this approach, materials are modeled as interacting particles confined to a finite range (the horizon), and the governing equations take an integral form without spatial derivatives. Therefore, PD is well-suited for fracture problems with bond-based [13,14] and state-based [15,16] formulations. So far, the PD approach has effectively addressed various static and dynamic fracture cases, including damage in fibers [17] and membranes [18], the failure analysis of composites [19,20], crack branching in brittle materials [21,22,23], and the dynamic fracture of superconductors [24,25,26]. In addition, PD methods coupled with the FEM have also been presented [27,28,29].

Once stress intensity factors (SIFs) are determined, the static and dynamic fracture behavior of cracked bodies can be anticipated. Hence, precise SIF determination is essential for investigating crack propagation. Classical linear elastic fracture mechanics, pioneered by Griffith [30] and later formalized by Irwin [31] and Rice [32], provides a framework for analyzing cracked bodies using SIFs, energy release rates, and the J-integral. With a proper integral contour, one can compute the strain energy release rate and subsequently obtain the SIF. Yau [33] and Stern [34] established an interaction integral based on the J-integral to evaluate mixed-mode SIFs in 2D cracked plates.

In the PD literature, studies on SIFs within the context of classical fracture mechanics remain limited. Hu [35] rigorously derived the J-integral formulation in bond-based peridynamics via the crack infinitesimal virtual extension method. Breitenfeld [36] proposed state-based peridynamics to evaluate numerical accuracy near the crack front. Panchadhara [37] introduced a post-processing method to extract SIFs from PD data. Le [38] proposed a method for correcting surface effects in PD. A new approach combining the displacement extrapolation method and peridynamic theory is presented by Zhu [39] to evaluate stress intensity factors. Zhang [40] developed a peridynamic virtual crack extension method to determine the energy release rate, where the virtual crack for crack propagation is constructed using uniquely applied directional and virtual broken bonds. Stenström [41] reformulated the J-area integral solely in terms of displacement variables, providing an alternative pathway for calculating the J-integral within the peridynamic framework. Le [42] established a simple computational scheme based on the virial theorem, using nodal tensile stresses to compute SIFs. However, the above studies focused on pure mode-I fractures, with few PD-based works addressing mixed-mode cases. Jung [43] used the PD J-integral to obtain mode-I/II SIFs for fatigue analysis. Zou [44] examined the J-integral under mixed-mode loads. A two-dimensional non-ordinary state-based peridynamics model based on three-body interaction potential is developed to simulate elastic brittle fracture behaviors for mixed-mode type fracture and crack dynamic branching cases [45]. Mirsayar [46] established a generalized approach to predict dynamic crack propagation by correlating the critical stretch with the critical strain energy release rate. The interaction integral has been used to extract mixed-mode DSIFs. Imachi [47] evaluated DSIFs for cracked solids with mixed-mode cracks. Dai [48] developed a PD shell model for mixed-mode SIFs under in-plane loading.

This paper presents an OSPD approach for DSIFs evaluation and crack growth simulation. The OSPD model was applied to 2D brittle cracked solids under static, quasi-static, and dynamic loading conditions. Mixed-mode DSIFs are extracted via the interaction integral method. The paper is structured as follows. Section 2 introduces the OSPD theory. Section 3 presents the interaction integral method. Section 4 analyzes several numerical examples, comparing PD results with analytical solutions and FEM results. Section 5 provides the conclusions.

## 2. Peridynamic Theory

### 2.1. 2D OSPD Model

**Figure 1:**
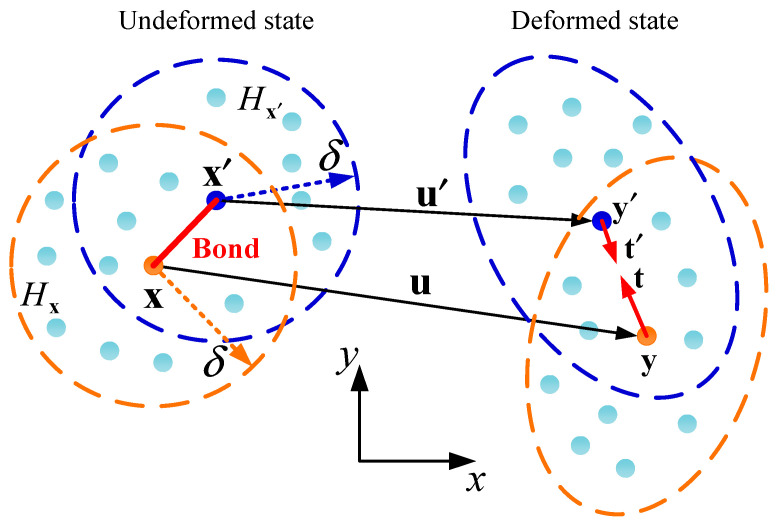
In the OSPD framework, blue dots denote material particles; blue and orange circles indicate the horizons of particles x and $x^{\prime }$, respectively.

PD uses particles and bonds to describe the materials. Particles interact only within a finite range referred to as the “horizon”; beyond it, no interaction occurs. In the OSPD theory, the equation of motion at point x takes the form of a nonlinear integro-differential equation [49]:

(1) $$\rho \left(x\right)\ddot{u}\left(x,t\right)=\int_{H_{x}}f\left(x,x^{\prime },u,u^{\prime },t\right)dV_{x^{\prime }}+b\left(x,t\right)$$

where $\rho \left(x\right)$, $\ddot{u}\left(x,t\right)$, and $b\left(x,t\right)$ denote the mass density, acceleration vector, and body force density of particle x, respectively. As shown in Figure 1, u and $u^{\prime }$ are the displacement of particles x and $x^{\prime }$. In the deformed state, the positions of x and $x^{\prime }$ are described by the vectors y and $y^{\prime }$. The region $H_{x}$ defines the neighborhood of particle x within the horizon δ, typically taken as a circle centered at x in 2D problems. The force function f can be derived as [50]:

(2) $$f=t\left(\eta ,\xi ,t\right)-t^{\prime }\left(\eta ,\xi ,t\right)$$

Here, $\xi =x^{\prime }-x$ and $\eta =u^{\prime }-u$ are the relative position vector and relative displacement vector, respectively. As illustrated in Figure 1, the force density vector t at particle x depends on all particles inside $H_{x}$. Similarly, particle $x^{\prime }$ is also affected by the deformation of points within its own family $H_{x^{\prime }}$.

The force density vector can be rewritten as [51]:

(3) $$t=2\delta \left\{\frac{\Lambda }{\left|\xi \right|}ad\theta_{\left(x\right)}+b\mu s\right\}\times \frac{\xi +\eta }{\left|\xi +\eta \right|}$$

The failure parameter μ is a factor, and the discrete form of the dilatation $\theta_{\left(x\right)}$ is

(4) $$\theta_{\left(x\right)}=d\delta \sum_{x^{\prime }=1}^{N}\Lambda \mu sV_{x^{\prime }}$$

The peridynamic parameters a, *d*, and b are determined from isotropic expansion and simple shear. Under two-dimensional plane strain conditions with a unit thickness assumption, the peridynamic parameters are given as [52]:

(5) $$a=\frac{\left(4v-1\right)E}{4(1-2v)(1+v)},\;b=\frac{3E}{(1+v)\pi \delta^{4}},\;d=\frac{2}{\pi \delta^{3}}$$

where E and v represent the Young’s modulus and Poisson’s ratio. The stretch s between particles x and $x^{\prime }$ is

(6) $$s=\frac{\left|\xi +\eta \right|-\left|\xi \right|}{\left|\xi \right|}$$

The scalar parameter Λ is defined as [51]:

(7) $$\Lambda =\left(\frac{\xi +\eta }{\left|\xi +\eta \right|}\right)\cdot \left(\frac{\xi }{\left|\xi \right|}\right)$$

In order to compute stress components in PD, a collapsed peridynamic stress tensor field is introduced [53]:

(8) $$\sigma =\int_{H_{x}}t⊗\xi dV_{x^{\prime }}$$

By combining Equations (2)–(4), the force function f can be rewritten as:

(9) $$f=2\delta \left\{\frac{\Lambda }{\left|\xi \right|}ad\left(\theta_{\left(x\right)}+\theta_{\left(x^{\prime }\right)}\right)+2b\mu s\right\}\times \frac{\xi +\eta }{\left|\xi +\eta \right|}$$

The parameter μ, which denotes bond breakage, is defined as [51]:

(10) $$\mu \left(\xi ,t\right)=\left\{\begin{array}{c} 1\\ 0 \end{array}\right.\begin{array}{c} s<s_{c},\\ \;\mathrm{otherwise} \end{array}$$

where $s_{c}$ represents the critical stretch of the bond which is related to the fracture properties of the material. For an isotropic material, $s_{c}$ and fracture toughness $K_{IC}$ satisfy [51]:

(11) $$\frac{K_{IC}^{2}\left(1-v^{2}\right)}{E}=\left(b\delta^{5}+\frac{8}{9}ad^{2}\delta^{7}\right)s_{c}^{2}$$

Local damage is defined as a fraction of broken bonds relative to all interactions:

(12) $$\varphi \left(x,t\right)=1-\frac{\int_{H_{x}}\mu \left(\xi ,t\right)dV_{\left(x^{\prime }\right)}}{\int_{H_{x}}dV_{\left(x^{\prime }\right)}}$$

The value of local damage varies from 0 and 1, where 1 means complete breakage at the material point and 0 means no damage.

### 2.2. Numerical Implementation

Kilic and Madenci [54] extended an adaptive dynamic relaxation approach to peridynamics framework for quasi-static problems in which the damping coefficient is updated at each iteration step according to the current deformation state. The PD motion equation with local damping is given by [55]:

(13) $$\rho \left(x\right)\ddot{u}\left(x,t\right)+C\dot{u}\left(x,t\right)=\int_{H_{x}}f\left(x,x^{\prime },u,u^{\prime },t\right)dV_{x^{\prime }}+b\left(x,t\right)$$

where C is a positive real constant. In the discretized peridynamic model, the computational domain is represented by uniformly spaced particles with a spacing of Δx. For each particle $x_{\left(k\right)}$, its family $H_{x_{\left(k\right)}}$ consists of all particles $x_{\left(j\right)}$ within a horizon δ=3Δx. To handle boundary effects, particles near the computational boundary are assigned a reduced family volume, following the approach in [51]. Therefore, the motion equation is discretized as:

(14) $$\rho {\ddot{u}}_{\left(k\right)}^{n}+C{\dot{u}}_{\left(k\right)}^{n}=L_{u}\left(x_{\left(k\right)}^{n},t^{n}\right)+b_{\left(k\right)}^{n}$$

where $L_{u}\left(x_{\left(k\right)}^{n},t^{n}\right)=\sum_{j=1}^{N_{p}}\left[t_{\left(k)(j\right)}^{n}-t_{\left(j)(k\right)}^{n}\right]V_{\left(j\right)}$. $u_{\left(k\right)}^{n}$ and $b_{\left(k\right)}^{n}$ are the displacement and body force density of particle $x_{\left(k\right)}$ at *n*-th time. $t_{\left(k)(j\right)}^{n}$ is the force density vector exerted on particle $x_{\left(k\right)}$ by particle $x_{\left(j\right)}$, and $V_{\left(j\right)}$ is the volume of $x_{\left(j\right)}$. $N_{P}$ is the number of points in the family of $x_{\left(k\right)}$. The explicit central difference scheme gives the acceleration and velocity as:

(15) $${\ddot{u}}_{\left(k\right)}^{n}=\frac{u_{\left(k\right)}^{n+1}-2u_{\left(k\right)}^{n}+u_{\left(k\right)}^{n-1}}{\Delta t^{2}},\;{\dot{u}}_{\left(k\right)}^{n}=\frac{u_{\left(k\right)}^{n+1}-u_{\left(k\right)}^{n-1}}{2\Delta t}$$

where Δt denotes the time interval. Thus, the displacement at time n+1 is given by [55]:

(16) $$u_{\left(k\right)}^{n+1}=\frac{2\rho }{\Delta t^{2}\left(\rho /\Delta t^{2}+C/2\Delta t\right)}u_{\left(k\right)}^{n}-\frac{\left(\rho /\Delta t^{2}-C/2\Delta t\right)}{\left(\rho /\Delta t^{2}+C/2\Delta t\right)}u_{\left(k\right)}^{n-1}+\frac{\left[L_{u}+b_{\left(k\right)}^{n}\right]}{\left(\rho /\Delta t^{2}+C/2\Delta t\right)}$$

Three types of problems are considered: static elastic deformation, quasi-static, and dynamic failure. For static problems, the body force density $b_{\left(k\right)}^{n}$ is time-independent and the local damping C is applied. For quasi-static problems, $b_{\left(k\right)}^{n}$ increases linearly with time, and C is also applied. For dynamic problems, $b_{\left(k\right)}^{n}$ is time-independent and C is set to zero. All constitutive laws and numerical schemes were coded in Fortran using a two-dimensional parallel code.

## 3. Interaction Integral Method

**Figure 2:**
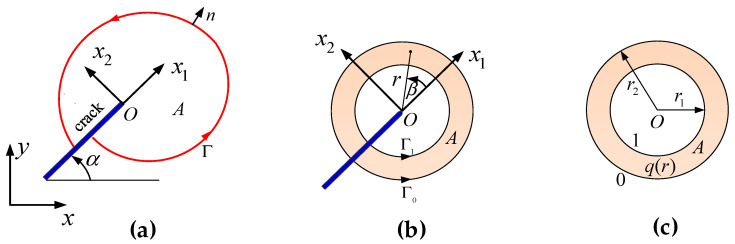
(**a**) Areas for J-integral; (**b**) equivalent domain integral; (**c**) weight function.

DSIFs describe the stress field near the crack front and are useful for dynamic fracture analysis [56]. The interaction integral efficiently computes SIFs within the OSPD framework. In this section, the interaction integral extracts mixed-mode DSIFs from the J-integral, which is given by [32]:

(17) $$J=\int_{\Gamma }\left(W\delta_{1j}-\sigma_{ij}u_{i,1}\right)n_{j}ds$$

where W is the strain energy density given by $W=\sigma_{ij}\varepsilon_{ij}/2$. $n_{j}$ is the outward normal vector to the contour Γ, as shown in Figure 2a. A global coordinate system $\left(x,y\right)$ is defined for the material, while a local coordinate system $\left(x_{1},x_{2}\right)$ is established at the crack-tip with its $x_{1}$-axis oriented along the crack direction. The angle between the two coordinate systems is denoted by α. The area A represents a representative region around the crack-tip. In this section, the indices $\left(i,j\right)$ range from 1 to 2. The comma in index notation denotes partial differentiation with respect to the corresponding spatial coordinate. For example, the partial derivative of the displacement $u_{2}$ (in the $x_{2}$-direction) with respect to $x_{1}$ is denoted as $u_{2,1}$, where the indices i and j each take the value 1 and 2, respectively. Assuming the crack faces are traction-free, application of the divergence theorem to Equation (17) leads to the equivalent domain integral (EDI) form of the J-integral as follows [57]:

(18) $$J=\int_{A}\left(\sigma_{ij}u_{i,1}-W\delta_{1j}\right)q_{,j}dA+\int_{A}{\left(\sigma_{ij}u_{i,1}-W\delta_{1j}\right)}_{,j}qdA$$

where q is a weight function that varies from q=1 on $\Gamma_{1}$ to q=0 on $\Gamma_{0}$ (see Figure 2b). The polar coordinates of an arbitrary point within region A are denoted as $\left(r,\beta \right)$. The weight function q used in this paper is expressed as follows:

(19) $$q\left(r\right)=1-\left(r-r_{1}\right)/\left(r_{2}-r_{1}\right)$$

where $r_{1}$ and $r_{2}$ represent the inner and outer radii of the area integral domain A, respectively, as shown in Figure 2c.

The Equation (18) is modified as [58]:

(20) $$J=\int_{A}\left(\sigma_{ij}u_{i,1}-W\delta_{1j}\right)q_{,j}dA+\int_{A}\sigma_{ij,j}u_{i,1}qdA$$

Using the following relationship:

(21) $$\begin{array}{l} W_{,1}=\frac{1}{2}\sigma_{ij,1}\varepsilon_{ij}+\frac{1}{2}\sigma_{ij}\varepsilon_{ij,1},\\ {\left(\sigma_{ij}u_{i,1}\right)}_{,j}=\sigma_{ij,j}u_{i,1}+\sigma_{ij}\varepsilon_{ij,1},\\ \sigma_{ij,1}\varepsilon_{ij}={\left(C_{ijkl}\varepsilon_{kl}\right)}_{,1}\varepsilon_{ij}=C_{ijkl,1}\varepsilon_{kl}\varepsilon_{ij}+\sigma_{ij}\varepsilon_{ij,1}, \end{array}$$

where $C_{ijkl}$ is the elasticity tensor, and it satisfies $C_{ijkl,1}=0$ under the assumption of an isotropic material. It should be noted that $\sigma_{ij,j}=0$ for static elastic problem, while $\sigma_{ij,j}=\rho {\ddot{u}}_{i}$ for dynamic analysis.

**Figure 3:**
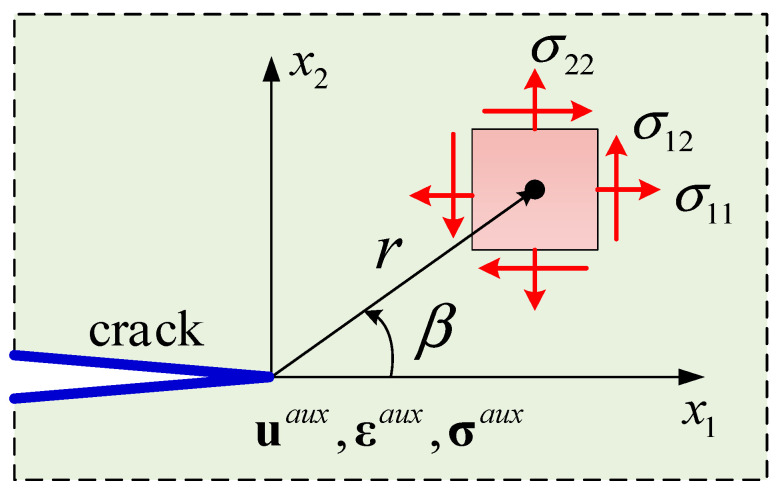
Auxiliary field: $\left(x_{1},x_{2}\right)$ and $\left(r,\beta \right)$ are the local and polar coordinates.

In the interaction integral method, the actual state $\left(\sigma_{ij}^{\mathrm{act}},\varepsilon_{ij}^{\mathrm{act}},u_{i}^{\mathrm{act}}\right)$ and the auxiliary state $\left(\sigma_{ij}^{\mathrm{aux}},\varepsilon_{ij}^{\mathrm{aux}},u_{i}^{\mathrm{aux}}\right)$ are required during the calculation. Figure 3 presents the auxiliary stress field near the crack-tip as [44]:

(22) $$\begin{array}{l} \sigma_{11}^{\mathrm{aux}}=\frac{K_{I}^{\mathrm{aux}}}{\sqrt{2\pi r}}\cos\frac{\beta }{2}\left(1-\sin\frac{\beta }{2}\sin\frac{3\beta }{2}\right)-\frac{K_{II}^{\mathrm{aux}}}{\sqrt{2\pi r}}\sin\frac{\beta }{2}\left(2+\cos\frac{\beta }{2}\cos\frac{3\beta }{2}\right)\\ \sigma_{22}^{\mathrm{aux}}=\frac{K_{I}^{\mathrm{aux}}}{\sqrt{2\pi r}}\cos\frac{\beta }{2}\left(1+\sin\frac{\beta }{2}\sin\frac{3\beta }{2}\right)+\frac{K_{II}^{\mathrm{aux}}}{\sqrt{2\pi r}}\sin\frac{\beta }{2}\cos\frac{\beta }{2}\cos\frac{3\beta }{2}\\ \sigma_{12}^{\mathrm{aux}}=\frac{K_{I}^{\mathrm{aux}}}{\sqrt{2\pi r}}\sin\frac{\beta }{2}\cos\frac{\beta }{2}\cos\frac{3\beta }{2}+\frac{K_{II}^{\mathrm{aux}}}{\sqrt{2\pi r}}\cos\frac{\beta }{2}\left(1-\sin\frac{\beta }{2}\sin\frac{3\beta }{2}\right) \end{array}$$

and the corresponding auxiliary displacement fields are:

(23) $$\begin{array}{l} u_{1}^{\mathrm{aux}}=\frac{\left(1+v\right)}{E}\sqrt{\frac{r}{2\pi }}\left[K_{I}^{\mathrm{aux}}\cos\frac{\beta }{2}\left(k_{\mathrm{tip}}-\cos\beta \right)+K_{II}^{\mathrm{aux}}\sin\frac{\beta }{2}\left(k_{\mathrm{tip}}+2+\cos\beta \right)\right]\\ u_{2}^{\mathrm{aux}}=\frac{\left(1+v\right)}{E}\sqrt{\frac{r}{2\pi }}\left[K_{I}^{\mathrm{aux}}\sin\frac{\beta }{2}\left(k_{\mathrm{tip}}-\cos\beta \right)-K_{II}^{\mathrm{aux}}\cos\frac{\beta }{2}\left(k_{\mathrm{tip}}-2+\cos\beta \right)\right] \end{array}$$

where $k_{\mathrm{tip}}=3-4v$ for plane strain evaluated at crack-tip. $K_{I}^{\mathrm{aux}}$ and $K_{II}^{\mathrm{aux}}$ denote the auxiliary SIFs for mode I and mode II.

The auxiliary strain fields are expressed as:

(24) $$\varepsilon_{ij}^{\mathrm{aux}}=\frac{1}{2}\left(u_{i,j}^{\mathrm{aux}}+u_{j,i}^{\mathrm{aux}}\right)$$

By linearly superimposing the actual and auxiliary field in Equation (20), the J-integral for a third equilibrium state is obtained as [32]:

(25) $$\begin{array}{ll} J= & \int_{A}\left\{\left(\sigma_{ij}^{\mathrm{act}}+\sigma_{ij}^{\mathrm{aux}}\right)\left(u_{i,1}^{\mathrm{act}}+u_{i,1}^{\mathrm{aux}}\right)-\frac{1}{2}\left(\sigma_{ik}^{\mathrm{act}}+\sigma_{ik}^{\mathrm{aux}}\right)\left(\varepsilon_{ik}^{\mathrm{act}}+\varepsilon_{ik}^{\mathrm{aux}}\right)\delta_{1j}\right\}q_{,j}dA\\ & +\int_{A}\left\{\left(\sigma_{ij,j}^{\mathrm{act}}+\sigma_{ij,j}^{\mathrm{aux}}\right)\left(u_{i,1}^{\mathrm{act}}+u_{i,1}^{\mathrm{aux}}\right)\right\}qdA=J^{\mathrm{act}}+J^{\mathrm{aux}}+M \end{array}$$

where $J^{\mathrm{act}}$ and $J^{\mathrm{aux}}$ are the J-integrals for the actual and auxiliary fields, respectively. M is their interaction integral, defined as:

(26) $$\begin{array}{ll} M= & \int_{A}\left\{\left(\sigma_{ij}^{\mathrm{act}}u_{i,1}^{\mathrm{aux}}+\sigma_{ij}^{\mathrm{aux}}u_{i,1}^{\mathrm{act}}\right)-\frac{1}{2}\left(\sigma_{ik}^{\mathrm{act}}\varepsilon_{ik}^{\mathrm{aux}}+\sigma_{ik}^{\mathrm{aux}}\varepsilon_{ik}^{\mathrm{act}}\right)\delta_{1j}\right\}q_{,j}dA\\ & +\int_{A}\left(\sigma_{ij,j}^{\mathrm{act}}u_{i,1}^{\mathrm{aux}}+\sigma_{ij,j}^{\mathrm{aux}}u_{i,1}^{\mathrm{act}}\right)qdA \end{array}$$

However, the actual stresses and displacements obtained from Equations (8) and (16) are expressed in the global coordinate system $\left(x,y\right)$. Their transformation to the local coordinate system $\left(x_{1},x_{2}\right)$ is accomplished using the following relationship:

(27) $$\begin{array}{l} {\left[\sigma^{\mathrm{act}}\right]}_{\left(x_{1},x_{2}\right)}=\left[R_{\left(\alpha \right)}\right]{\left[\sigma \right]}_{\left(x,y\right)}{\left[R_{\left(\alpha \right)}\right]}^{T}\\ {\left[u^{\mathrm{act}}\right]}_{\left(x_{1},x_{2}\right)}=\left[R_{\left(\alpha \right)}\right]{\left[u\right]}_{\left(x,y\right)} \end{array}$$

where $\left[R_{\left(\alpha \right)}\right]$ denotes the transformation matrix between two coordinate systems. Meanwhile, the auxiliary stresses, displacements, and weight function are defined in the polar coordinate system $\left(r,\beta \right)$. The corresponding differentials with respect to the local coordinates are given by:

(28) $$\begin{array}{l} \frac{\partial \left(\sigma^{\mathrm{aux}},u^{\mathrm{aux}},q\right)}{\partial x_{1}}=\frac{\partial \left(\sigma^{\mathrm{aux}},u^{\mathrm{aux}},q\right)}{\partial r}\cos\beta +\frac{\partial \left(\sigma^{\mathrm{aux}},u^{\mathrm{aux}},q\right)}{\partial \beta }\left(\frac{-\sin\beta }{r}\right)\\ \frac{\partial \left(\sigma^{\mathrm{aux}},u^{\mathrm{aux}},q\right)}{\partial x_{2}}=\frac{\partial \left(\sigma^{\mathrm{aux}},u^{\mathrm{aux}},q\right)}{\partial r}\sin\beta +\frac{\partial \left(\sigma^{\mathrm{aux}},u^{\mathrm{aux}},q\right)}{\partial \beta }\left(\frac{\cos\beta }{r}\right) \end{array}$$

The interaction integral relates to the mixed-mode SIFs of the actual and auxiliary fields as follows [58]:

(29) $$M=\frac{2\left(1-v^{2}\right)}{E}\left(K_{I}^{\mathrm{act}}K_{I}^{\mathrm{aux}}+K_{II}^{\mathrm{act}}K_{II}^{\mathrm{aux}}\right)$$

By properly selecting the auxiliary SIFs $K_{I}^{\mathrm{aux}}$ and $K_{II}^{\mathrm{aux}}$, the actual SIFs are obtained separately:

(30) $$\begin{array}{l} K_{I}^{\mathrm{act}}=\frac{E}{2\left(1-v^{2}\right)}M\;\left(K_{I}^{\mathrm{aux}}=1,K_{II}^{\mathrm{aux}}=0\right)\\ K_{II}^{\mathrm{act}}=\frac{E}{2\left(1-v^{2}\right)}M\;\left(K_{I}^{\mathrm{aux}}=0,K_{II}^{\mathrm{aux}}=1\right) \end{array}$$

## 4. Numerical Results and Discussion of DSIFs

In all numerical examples, the normalized SIFs and DSIFs are employed as follows:

(31) $$K_{i}=K_{i}^{\mathrm{act}}/\left(\sigma_{0}\sqrt{\pi a_{c}}\right),\;\left(i=I,II\right)$$

where $\sigma_{0}$ is the applied stress on the cracked brittle solid, $a_{c}$ is the initial crack length, and the horizon satisfied δ=3Δx for every case.

The DSIFs and crack propagation were investigated for mode I and mixed-mode cracked plates under static, quasi-static, and dynamic conditions. Additionally, the effects of holes on the DSIFs and crack propagation paths were considered. The numerical results obtained from PD theory were validated against available analytical predictions and FEM results.

### 4.1. Mode I Cracked Plate Under Static and Quasi-Static Analyses

**Figure 4:**
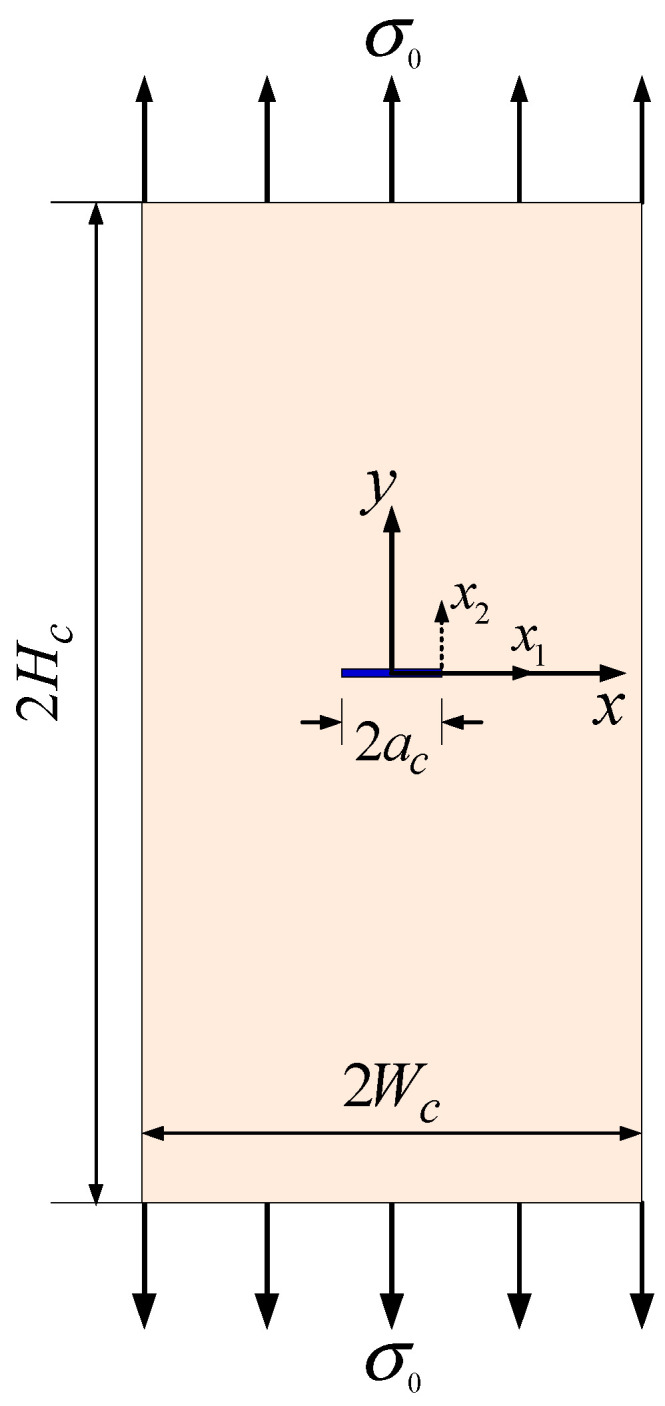
A finite plate with a center crack.

A finite plate (see Figure 4) with width $2W_{c}=200\;\mathrm{mm}$, height $2H_{c}=400\;\mathrm{mm}$, and a center crack of length $2a_{c}=40\;\mathrm{mm}$ is considered. The material has Young’s modulus E=200 GPa and Poisson’s ratio v=0.25. The particle distance Δx is 1 mm, and the magnitude of the applied stress is $\sigma_{0}=100\;\mathrm{MPa}$. This classical mode I fracture problem has its SIF determined from a periodic array of cracks in an infinite plate. The reference dimensionless SIF for this case is given as follows [59]:

(32) $$K_{I}^{c}=\sqrt{\sec\frac{\pi a_{c}}{2W_{c}}}\left[1-0.025{\left(\frac{a_{c}}{W_{c}}\right)}^{2}+0.06{\left(\frac{a_{c}}{W_{c}}\right)}^{4}\right]=1.0245$$

**Figure 5:**
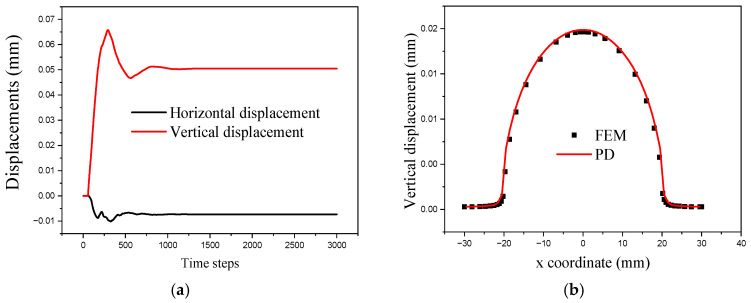
(**a**) Convergence of displacement at a monitored point; (**b**) Comparison of the vertical displacement from PD and FEM.

Figure 5a shows the displacement versus time step for a particle at x=50.5 mm, y=100.5 mm, where the local damping coefficient is set to $C=2\times 10^{8}$. The displacement of the monitored point converges to a steady-state value after approximately 2000 time steps. No oscillations or abrupt changes are observed beyond 2000 time steps, confirming that the introduction of local damping effectively stabilizes the computation without affecting the static solution. Figure 5b compares the vertical displacement distribution along the crack surface obtained from the FEM and the converged displacement solution from the peridynamic model. The displacement decreases rapidly from the crack center toward both crack tips. The two numerical approaches are in excellent agreement, confirming that the peridynamic simulation accurately captures the local deformation fields in a tensile-loaded cracked plate.

**Figure 6:**
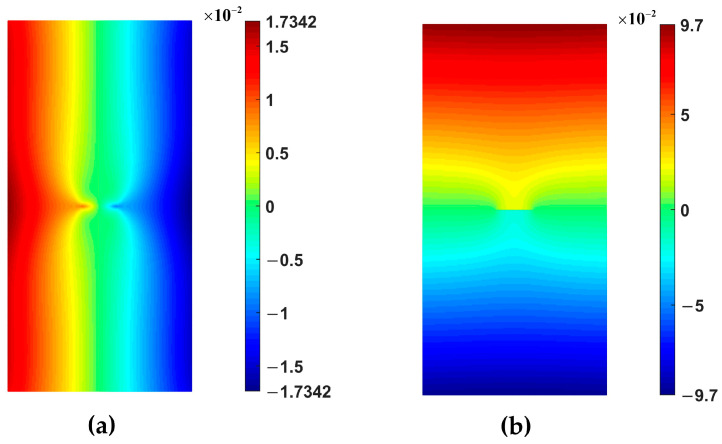
Plate displacement contours (unit: mm): (**a**) horizontal; (**b**) vertical. The maximum and minimum values are indicated on the color bars.

The displacement contours of the cracked plate under static conditions are shown in Figure 6. The results reveal a symmetric displacement distribution about the crack plane. The maximum horizontal and vertical displacements obtained from the PD simulation are $1.7342\times 10^{-2}\;\mathrm{mm}$ and $9.7\times 10^{-2}\;\mathrm{mm}$, respectively, demonstrating excellent agreement with the FEM results of $1.7279\times 10^{-2}\;\mathrm{mm}$ and $9.707\times 10^{-2}\;\mathrm{mm}$. It should be noted that the displacement patterns are consistent with the classical elastic fracture mechanics predictions, which verifies the accuracy and validity of the peridynamic solution.

**Figure 7:**
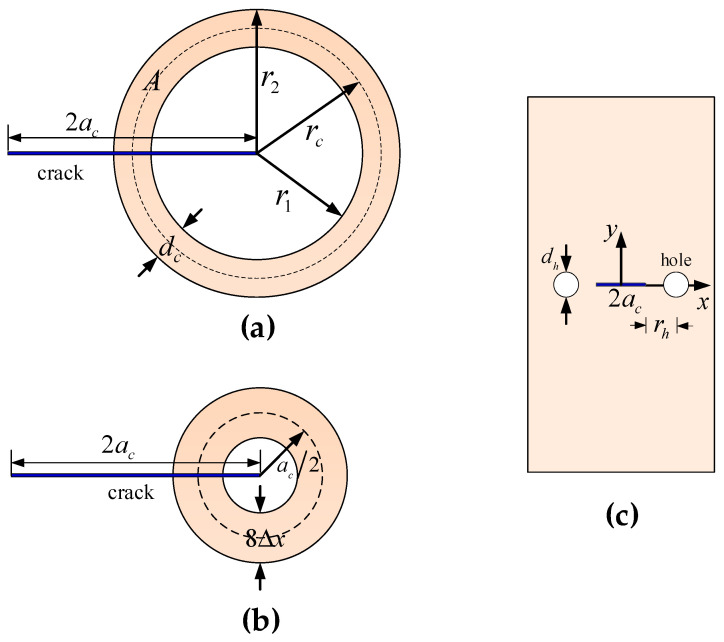
(**a**) Definition of the integration domain; (**b**) choice of the integration domain; (**c**) schematic diagram of a finite plate containing a center crack and two circular holes.

**Figure 8:**
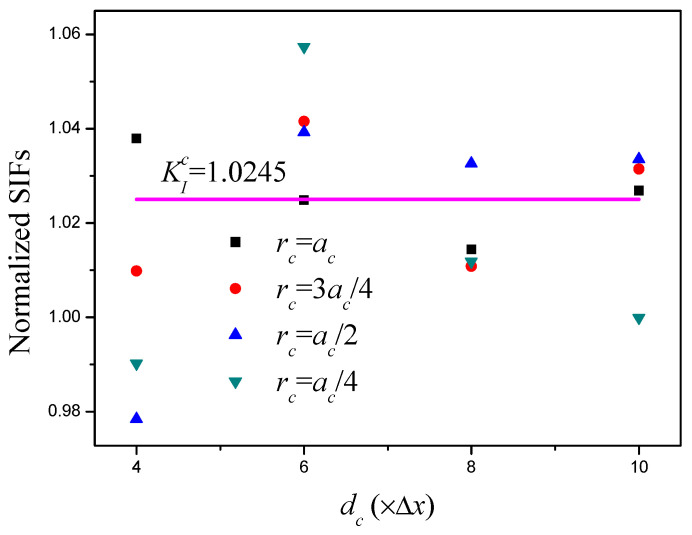
Normalized SIFs for different integration domain thicknesses $d_{c}$ and radii $r_{c}$.

The calculation of SIFs requires the definition of an integration domain. Figure 7a illustrates the integration domain for the interaction integral, which is characterized by the thickness $d_{c}$ and the radius $r_{c}$ of area A, with $d_{c}=r_{2}-r_{1}$ and $r_{c}=\left(r_{2}+r_{1}\right)/2$. Figure 8 presents the normalized SIFs obtained with different combinations of the integration domain thickness $d_{c}$ and radius $r_{c}$. The theoretical value for this classical mode I fracture problem is 1.0245. The application of PD theory may introduce inaccuracies due to the skin effect, which arises from surface effects and non-local horizon interactions involving particles at the body’s boundary. This error becomes substantial when the integration domain of Equation (26) is small. Furthermore, discretizing the annular integration domain into particles causes slight variations in the effective domain area as $d_{c}$ changes, leading to minor oscillations in the computed SIFs. For a given thickness, increasing the radius generally improves the accuracy of the SIF. Considering both accuracy and computational efficiency, the parameters $r_{c}=a_{c}/2$ and $d_{c}=8\Delta x$ are adopted for the subsequent analyses. The SIF calculated with the selected thickness and radius is 1.0326, with an error of less than 1%. The corresponding integration domain is illustrated in Figure 7b.

**Figure 9:**
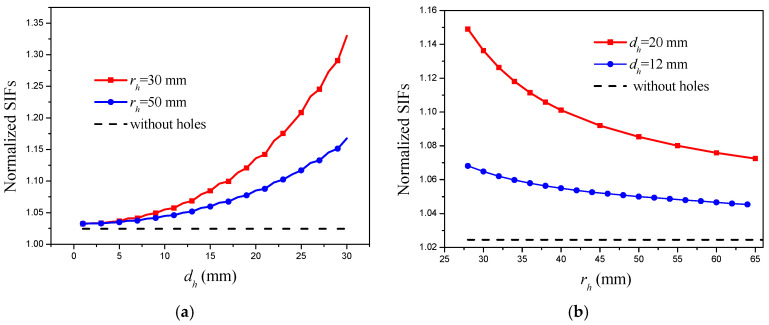
Variation in normalized SIFs with (**a**) the diameter $d_{h}$ and (**b**) the distances $r_{h}$.

Figure 7c illustrates the plate containing a center crack and two circular holes, where $d_{h}$ is the hole diameter and $r_{h}$ is the distance from the hole center to the crack tip. Both the diameter and the position of the hole are found to influence the SIF at the crack tip. The effects of a circular hole on SIFs under static loading are shown in Figure 9.

Figure 9a presents the variation in normalized SIFs with hole diameter $d_{h}$ for different distances $r_{h}$, while Figure 9b shows the variation in normalized SIFs with $r_{h}$ for different $d_{h}$. The SIFs vary nonlinearly with both $d_{h}$ and $r_{h}$. As shown in Figure 9a, when the diameter is small, the SIFs are close to those of the cracked plate without holes. However, once the hole diameter exceeds 10 mm, a noticeable increase in the SIFs is observed, particularly for holes located closer to the crack tip. As illustrated in Figure 9b, for holes located far from the crack tip, the normalized SIFs obtained from the PD simulation gradually approach those of a cracked plate without holes. A more rapid increase in SIFs is observed when the hole is closest to the crack tip, especially for larger hole diameters. Consequently, larger holes located closer to the crack tip result in higher SIFs, which may promote crack propagation.

**Figure 10:**
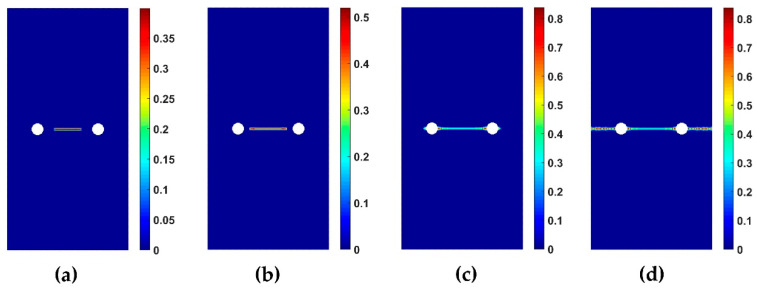
Damage maps in the cracked plate under different external stresses: (**a**) σ=93.23 MPa; (**b**) σ=94.3 MPa; (**c**) σ=95.63 MPa; (**d**) σ=97.37 MPa. White circles denote circular holes.

Figure 10 shows the damage contours reflecting the crack propagation paths in the plate. The normalized fracture toughness is 0.95, and the local damping coefficient is $8\times 10^{7}$. The diameter of the hole is 10 mm, and the distance from the hole center to the crack tip is 30 mm. Under quasi-static loading with an increment of 0.0167 MPa, several material bonds near the crack tip fail and local damage occurs when the external tensile stress reaches 93.23 MPa, as shown in Figure 10a. However, the particle system remains in equilibrium despite the broken bonds near the crack tip. When the external load increases to 94.3 MPa, the plate can no longer maintain equilibrium. Consequently, the crack propagates horizontally from the crack tip to the lateral hole, then passes through it and continues to propagate to both sides of the plate, as shown in Figure 10b–d.

### 4.2. Mixed-Mode Cracked Plate Under Static and Quasi-Static Analyses

**Figure 11:**
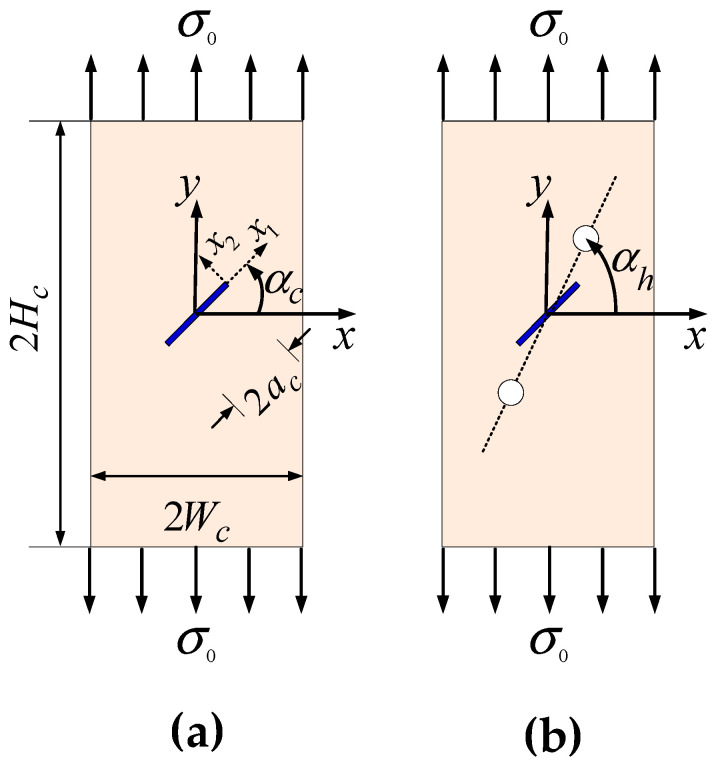
Geometry of rectangular plate: (**a**) with a center inclined crack, and (**b**) with a center inclined crack and two circular holes.

In contrast to the mode I crack in Section 4.1 (where only $K_{I}$ exists), the present example involves a mixed-mode crack characterized by both $K_{I}$ and $K_{II}$. A rectangular plate (see Figure 11a) of width $2W_{c}=5\;m$, height $2H_{c}=10\;m$, and a center inclined crack of length $2a_{c}=2\;m$ is considered. The crack inclination angle is set to $\alpha_{c}=\pi /4$. The material has Young’s modulus E=200 GPa and Poisson’s ratio v=0.3. The particle distance Δx is 25 mm, and the magnitude of the applied stress is $\sigma_{0}=100\;\mathrm{MPa}$.

In linear elastic fracture mechanics, the crack grows along the direction of maximum circumferential stress, given by [60]:

(33) $$K_{I}\sin\psi_{0}+K_{II}\left(3\cos\psi_{0}-1\right)=0$$

where $\psi_{0}$ denotes the fracture angle of the mixed-mode crack. To correspond to the fracture criterion for mode I cracks, the equivalent stress intensity factor for mixed-mode cracks is expressed as:

(34) $$K_{eq}=\cos\frac{\psi_{0}}{2}\left(K_{I}\cos^{2}\frac{\psi_{0}}{2}-\frac{3}{2}K_{II}\sin\psi_{0}\right)$$

Accordingly, the mixed-mode crack is considered to fail when its equivalent stress intensity factor $K_{eq}$ equals the material fracture toughness $K_{IC}$. The analytical normalized SIFs for static analysis are $K_{I}^{c}=0.5719$ and $K_{II}^{c}=0.5290$ [61]. The SIFs increase linearly with the applied load for quasi-static analysis.

**Figure 12:**
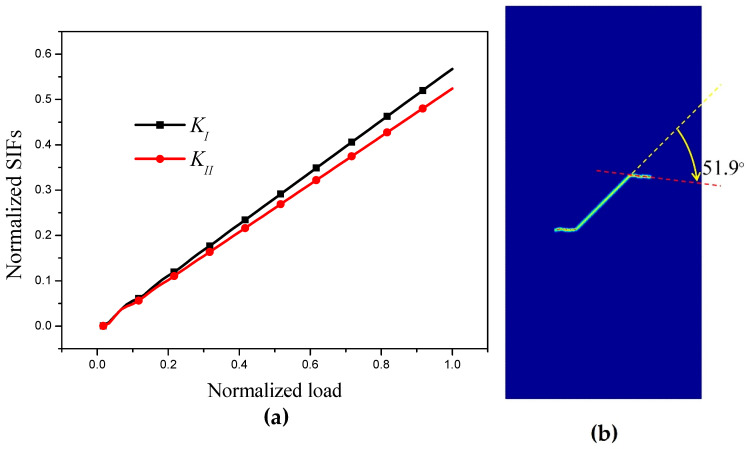
(**a**) Normalized SIFs under quasi-static loading; (**b**) comparison of crack propagation path obtained from PD simulation with theoretical solution. The yellow and red dashed lines represent the crack direction and its theoretical propagation direction, respectively.

Figure 12a shows the variation in the normalized SIFs with load under quasi-static loading, where both SIFs increase linearly with the applied load. The maximum values of $K_{I}$ and $K_{II}$ obtained from the PD simulation are 0.56756 and 0.52408, respectively, which closely match the static theoretical results of 0.5719 and 0.529. This linear relationship is consistent with the theoretical expectation for quasi-static mixed-mode fractures. Figure 12b compares the crack propagation path obtained from PD simulation with theoretical prediction. The red dashed line denotes the theoretical quasi-static crack propagation path predicted by the maximum circumferential stress criterion. For a crack inclination angle of 45°, Equation (33) predicts that the initial crack propagation direction makes an angle of −51.9° with respect to the crack orientation. According to Equation (34), the maximum value of the equivalent SIF is 0.9772. The normalized fracture toughness is determined based on the assumption that, for crack propagation to occur, it is set to three-quarters of the maximum equivalent SIF, which is taken as 0.7329. The numerical results show that the crack propagation agrees remarkably well with the theoretical prediction, further validating the accuracy of the proposed peridynamic approach.

**Figure 13:**
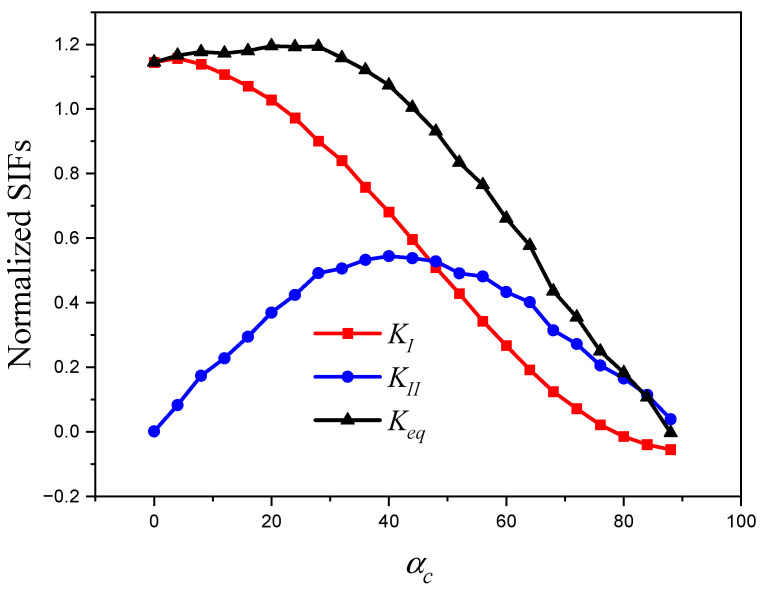
The effect of $\alpha_{c}$ on the SIFs for a center-inclined crack.

**Figure 14:**
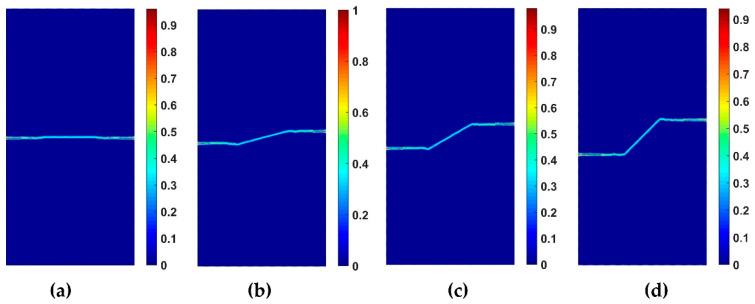
Damage maps for various inclination angles of a center-cracked rectangular plate: (**a**) 0°; (**b**) 15°; (**c**) 30°; (**d**) 45°.

Figure 13 shows the variation of $K_{I}$, $K_{II}$, and $K_{eq}$ with the crack inclination angle $\alpha_{c}$ under static loading. $K_{I}$ reaches its maximum value at $\alpha_{c}=0{}^{\circ}$, while the $K_{II}$ is zero. As the crack inclination angle increases, $K_{I}$ gradually decreases. $K_{II}$ first increases and then decreases, reaching its maximum near $\alpha_{c}=40{}^{\circ}$. The equivalent SIF $K_{eq}$ remains nearly constant at small inclination angles and then decreases significantly. Cracks with small inclination angles are dominated by mode I fractures, while those with moderate angles exhibit mixed-mode behavior. Highly inclined cracks experience low SIFs and are unlikely to propagate. Figure 14 presents the damage map in a rectangular plate containing a center inclined crack for different inclination angles under quasi-static loading. The crack initiation angle varies with the inclination angle of the pre-existing crack. However, as the applied load continues to increase, all cracks gradually propagate toward the direction perpendicular to the tensile load. Despite the distinct initial crack orientations, the resulting propagation paths of the four cracks are generally parallel to each other.

The effect of a circular hole on the SIFs and crack propagation path is investigated. As illustrated in Figure 11b, a rectangular plate with a 45° inclined center and two circular holes is considered. The hole has a diameter of 0.6 m and is located at a distance of 2 m from the crack center. The angular position of the hole is defined by the angle $\alpha_{h}$, which is measured between the line connecting the hole center to the crack center and the positive *x*-axis.

**Figure 15:**
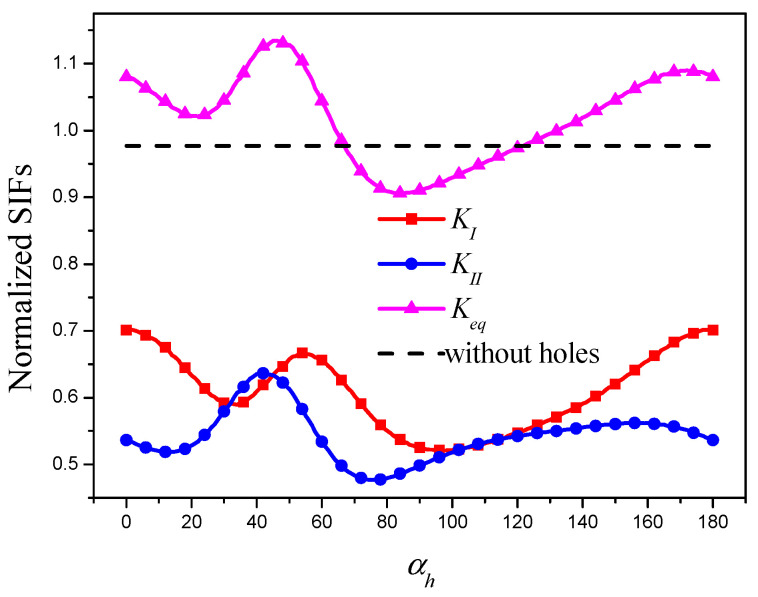
Variation in normalized SIFs with $\alpha_{h}$.

Figure 15 illustrates the variation in SIFs with the hole inclination angle $\alpha_{h}$, and the dashed line represents the static equivalent SIF for the plate without holes (0.9772). As shown in Figure 15, the hole effectively increases the peak value of the equivalent SIF. Furthermore, $K_{I}$ and $K_{II}$ exhibit distinctly different evolution patterns as $\alpha_{h}$ increases. The values of $K_{I}$ and $K_{II}$ are close to each other at $\alpha_{h}=45{}^{\circ}$. At this angle, the center of the hole is located on the extension line of the inclined crack, and the equivalent SIF reaches its maximum, indicating that the crack is most prone to unstable propagation under this configuration.

**Figure 16:**
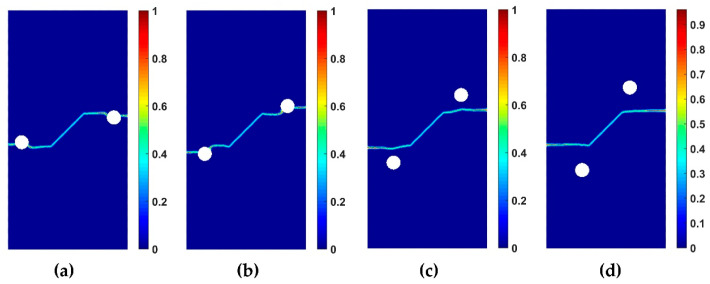
Damage maps for various hole inclination angles: (**a**) 15°; (**b**) 30°; (**c**) 45°; (**d**) 60°. White circles denote circular holes.

Figure 16 presents the crack propagation paths for a rectangular plate containing a 45° inclined center crack and two circular holes at four different angular positions: $\alpha_{h}=15{}^{\circ},\;30{}^{\circ},\;45{}^{\circ}$, and 60°. For $\alpha_{h}=15{}^{\circ},30{}^{\circ}$, and 45°, the crack initially propagates along its original direction and then gradually deviates toward the hole, as shown in Figure 16a–c. Once the crack tip reaches the hole surface, the crack passes through the hole and continues to propagate. It is observed that when the hole is located far from the crack propagation path, its influence on the crack path is negligible, and the crack propagates essentially along its own direction (see Figure 16d).

### 4.3. Mixed-Mode Cracked Plate Under Dynamic Fracture and Crack Branching

**Figure 17:**
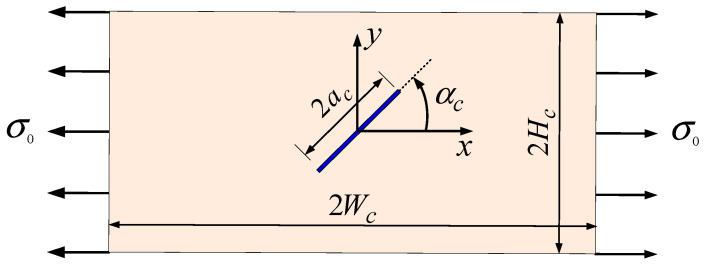
A rectangular plate contains a center inclined crack.

The specific geometric and material parameters in this section are adopted from the dynamic fracture benchmarks to enable a quantitative validation of the proposed PD approach. A rectangular plate (see Figure 17) of width $2W_{c}=60\;\mathrm{mm}$, height $2H_{c}=30\;\mathrm{mm}$, and a center inclined crack of length $2a_{c}=14.14\;\mathrm{mm}$ is considered. The plate is oriented with the crack aligned horizontally to facilitate the visualization of crack branching under dynamic loading. The material has Young’s modulus E=199.992 GPa, Poisson’s ratio v=0.3, and mass density $\rho =5000\mathrm{kg}/m^{3}$. A particle spacing Δx=0.15 mm is used, and the local damping is removed for dynamic analysis. An external stress of magnitude $\sigma_{0}=100\;\mathrm{MPa}$ is applied instantaneously with a step function. Before proceeding to the dynamic analysis, the static SIF of the structure is obtained. The normalized SIF values are calculated to be $K_{I}^{\mathrm{static}}=0.6$ and $K_{II}^{\mathrm{static}}=0.541$, which serve as a reference for the subsequent dynamic analysis.

**Figure 18:**
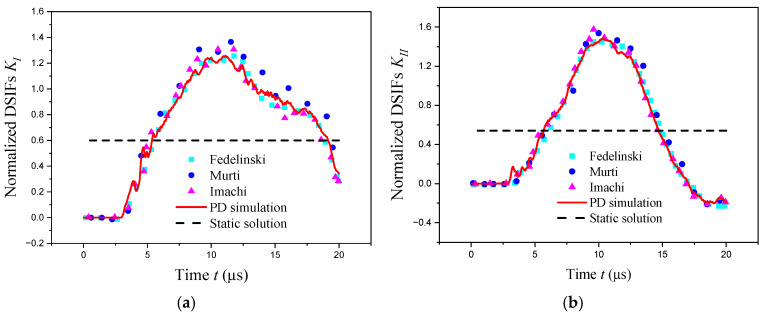
Comparison of the normalized DSIFs between the PD result and reference solutions: (**a**) $K_{I}$; (**b**) $K_{II}$.

**Figure 19:**
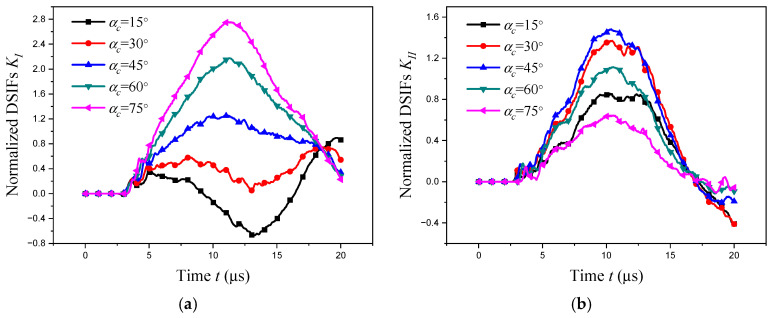
Normalized DSIFs for different crack inclination angles: (**a**) $K_{I}$; (**b**) $K_{II}$.

A comparison of the normalized DSIFs with those of Fedelinski [62], Murti [63], and Imachi [47] is shown in Figure 18. It is observed that the peak values under dynamic loading are higher than those of the static solution. The numerical results show good agreement with the reference data. DSIFs for five crack inclination angles are presented in Figure 19. The peak value of $K_{I}$ increases with the crack inclination angle. Among the five angles considered, the maximum peak value of $K_{II}$ occurs at $\alpha_{c}=45{}^{\circ}$.

**Figure 20:**
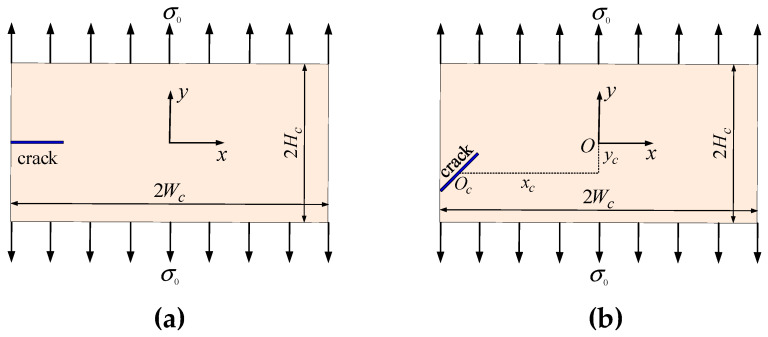
Rectangular plate with an edge crack: (**a**) straight crack; (**b**) inclined crack.

Dynamic brittle fracture experiments have consistently observed crack branching. In this study, the PD approach is demonstrated to be capable of modeling dynamic fracture and crack branching problems. The rectangular plate has the same geometric dimensions and material property as those in Figure 17, except that the crack length is 10 mm. As illustrated in Figure 20, both an edge straight crack and edge inclined crack are considered. The center of inclined crack is located at $\left(-x_{c},-y_{c}\right)$, where $x_{c}=26.454\;\mathrm{mm}$ and $y_{c}=3.5355\;\mathrm{mm}$. The critical stretch corresponding to the fracture toughness is assumed to be $s_{c}=5\times 10^{-4}$.

**Figure 21:**
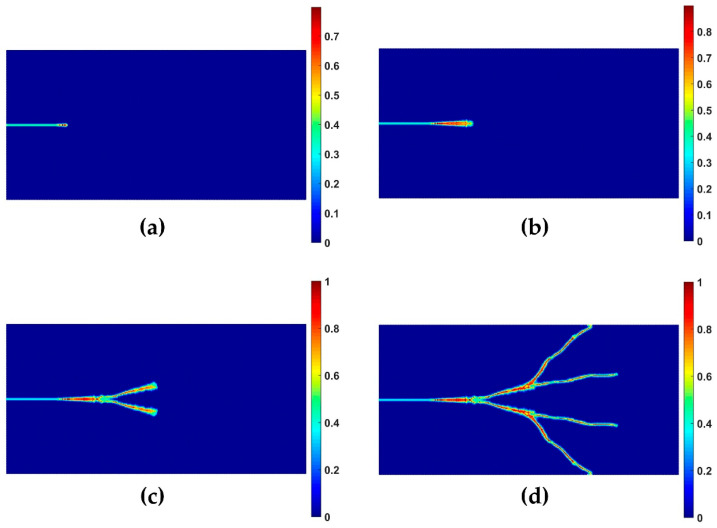
Dynamic fracture branching in an edge straight crack rectangular plate, damage maps for: (**a**) t=4.02 μs; (**b**) t=7.02 μs; (**c**) t=13.02 μs; (**d**) t=23.58 μs.

**Figure 22:**
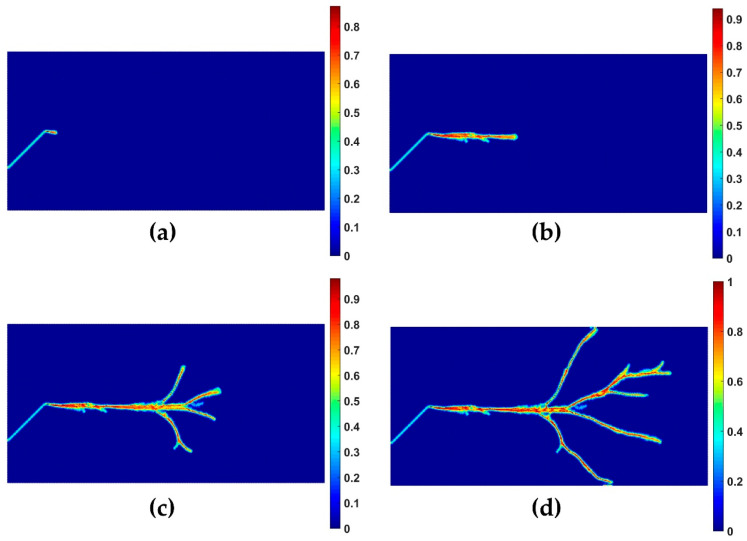
Dynamic fracture branching in an edge inclined crack rectangular plate, damage maps for: (**a**) t=4.02 μs; (**b**) t=10.62 μs; (**c**) t=17.82 μs; (**d**) t=23.82 μs.

Figure 21 illustrates the crack propagation and branching process in a rectangular plate with an external load of 23 MPa. Damage occurs at the crack tip (see Figure 21a), and the crack then propagates horizontally as is typical for a mode I crack (see Figure 21b). The primary crack branches successively and symmetrically as shown in Figure 21c,d. The two primary branches continue to grow until the plate boundary, and secondary branches begin to form. The observed branching behavior is qualitatively consistent with existing experimental studies [64,65,66], which validates the capability of the peridynamic approach for simulating dynamic fracture problems. Figure 22 presents damage maps of a rectangular plate with an edge-inclined crack. The crack begins to propagate at t=4.02 μs. In contrast to the horizontal propagation of the straight crack, the inclined crack deviates from its original orientation and deflects toward the horizontal direction, exhibiting mixed-mode fracture characteristics. This asymmetric branching is attributed to the asymmetric stress distribution at the crack tip. It is worth noting that some branches arrest after propagating a short distance, while others continue to grow until they approach the plate boundary. This phenomenon can be explained by the dynamic energy balance at the crack tip. Upon branching, the available strain energy release rate is distributed among the multiple crack tips. Branches that receive higher energy continue to propagate, whereas those with insufficient energy undergo arrest. Additionally, stress wave reflections from the plate boundaries cause temporary fluctuations in local SIFs. Asymmetrically oriented branches, such as those in the inclined crack case, experience non-uniform stress wave shielding and amplification, which further promotes arrest in certain directions.

## 5. Conclusions

In this study, an OSPD framework was presented for evaluating DSIFs and simulating crack propagation in brittle materials under static, quasi-static, and dynamic loading conditions. An interaction integral method was effectively incorporated into the PD theory to extract DSIFs for mode I and mixed-mode crack, and a local damping technique was employed to stabilize static and quasi-static solutions. The proposed numerical implementation was validated through comprehensive comparisons with theoretical predictions and finite element analyses.

The results demonstrated that the proposed PD approach accurately captured mode I and mixed-mode fracture characteristics. For static and quasi-static problems, the computed SIFs and displacement fields showed good agreement with classical fracture mechanics solutions. The influence of circular holes on SIFs and crack propagation paths was systematically examined. It was found that larger holes located closer to the crack tip result in higher SIFs, thereby promoting crack initiation and propagation. Furthermore, holes can deflect propagating cracks depending on their position. Under dynamic conditions, the DSIFs exhibited pronounced fluctuations, with peak values exceeding their static counterparts. The method successfully captured complex fracture phenomena, including crack branching under dynamic loading, where symmetric and asymmetric branching patterns were observed for straight and inclined edge cracks, respectively.

Beyond the benchmark problems, the proposed OSPD approach is particularly well-suited for investigating more complex fracture systems. These include dynamic fractures in heterogeneous materials such as composites or rocks with embedded inclusions, where classical methods often fail due to intricate crack-inclusion interactions, hydraulic fracturing in brittle solids, in which the coupling between fluid pressure and crack propagation remains challenging for traditional models, and fragmentation processes under high-strain-rate loading, such as ballistic impact or explosive loading, where PD naturally captures crack branching and coalescence. Continued development of peridynamic methods will open new avenues for addressing previously intractable fracture problems in engineering and materials science.

## Data Availability

The original contributions presented in this study are included in the article. Further inquiries can be directed to the corresponding authors.
